# What Directions Do We Look at Power from? Up-Down, Left-Right, and Front-Back

**DOI:** 10.1371/journal.pone.0132756

**Published:** 2015-07-10

**Authors:** Aitao Lu, Meichao Zhang, Yulan Shao, Yanping Yu, Shuang Zheng, Jing Ye, Hui Yi, Lu Wang

**Affiliations:** 1 Center for Studies of Psychological Application & School of Psychology, South China Normal University, Guangzhou, China; 2 Guangdong Key Laboratory of Mental Health and Cognitive Science, Guangzhou, China; 3 Guangdong Center of Mental Assistance and Contingency Technique for Emergency, Guangzhou, China; 4 Laboratory of Behavioral Science, Institute of Psychology, Chinese Academy of Sciences, Beijing, China; 5 University of Chinese Academy of Sciences, Beijing, China; Birkbeck, University of London, UNITED KINGDOM

## Abstract

Three experiments were carried out to investigate whether the the kinship concept had spatial representations along up-down (Experiment 1), left-right (Experiment 2), and front-back (Experiment 3) orientation. Participants identified the letter P or Q after judging whether kinship words were elder or junior terms. The results showed that participants responded faster to letters placed at the top, right side, and front following elder terms, and faster at the bottom, left side, and back following junior terms. The regression results further confirmed that these shifts of attention along up-down, right-left, and front-back dimensions in external space were uniquely attributed to the power construct embedded in the kinship concept, but not number or time. The results provide evidence for the multiple spatial representations in power, and can be explained by the theoretical construct of structural mapping.

## Introduction

The relationship between abstract concepts and concrete percepts has long been the subject of philosophical inquiry and psychological experimentation [1–3]. There is no doubt that abstract concepts and concrete percepts are intimately linked in the human mind, as demonstrated in many areas, e.g., power [4–6], valence [7], and morality [8]. According to conceptual metaphor theory, an abstract concept is metaphorically grounded in the concrete experience of physical domains [1]. For example, understanding the meaning of words and utterances has been found to engage the same neural and cognitive systems we use to perceive and interact with the physical world [9–10]. Perceptual characteristics of stimuli (e.g., their vertical position) could affect the speed with which words referring to abstract concepts (e.g., valence) are categorized [7]. Recently, focusing on the relational structure in metaphors (typically consisting of polar oppositions), Proctor and Cho [11] proposed a polarity account, which assumes that stimulus and response alternatives are coded as having positive (+polar endpoints) and negative (-polar endpoints) polarity along several dimensions, and their polarity correspondence is sufficient to produce mapping effects. That is, the structural overlap in polarities between perceptual locations and word meaning could explain the findings that words presented in metaphor congruent locations (e.g., positive words UP on the screen and negative words DOWN on the screen) are categorized faster than words presented in metaphor incongruent locations (e.g., positive words DOWN and negative words UP). It should be noted that the above two theories are particularly inconsistent in that the former explains the mapping between conceptual and perceptual domains in terms of the interference between concept and space due to the spatial representations in abstract concept, whereas the latter explains it in terms of overlapping polarities (i.e., stimulus-response mapping), without there being any overlap in conceptual representations.

So far, a large literature has examined patterns in how abstract concepts are mapped with concrete percepts. One is vertical mapping. For example, Meier and Robinson’s study [7] revealed that participants asked to classify words according to valence (i.e., positive or negative emotions) responded faster when positive items (e.g., love) were presented at the top of a screen rather than at the bottom, and vice versa for negative items (e.g., danger). People also judge a group's social power to be greater when these judgments are made at the top of a computer screen than when presented in the lower part of the screen [5]. Others have found evidence that morality-related terms [12], power-related terms [4], divinity-related terms [13], number-related terms [14–15], and time-related terms [16–19] all have a vertical orientation (moral, power, god, high number, and early—UP; immoral, weakness, devil, low number, and late—DOWN). These findings support the idea that conceptual processing (e.g., power, morality, divinity, number, and time) can elicit representations of verticality.

Another pattern is horizontal mapping. For example, people associate spatial position along the horizontal dimension with numerical magnitudes (the SNARC effect; spatial-numerical association of response codes) [20]. The SNARC effect indicates that larger numbers are responded to faster with the right hand, and smaller numbers are responded to faster with the left hand. An increasing body of evidence has shown that when adults engage in numerical processing, and even when numbers are irrelevant to the task at hand, their performance in behavioral tasks such as comparison, line bisection, and stimulus detection, exhibits spatially-related effects that are consistent with the hypothesis of a spontaneous mapping of numbers onto a horizontally-oriented space [20–21]. Moreover, this left-to-right axis also forms a basis for systems that represent time [22], and even the conception of events [23]. These findings support the idea that conceptual processing (e.g., number, time, and conception of events) can elicit representations of horizontality.

Reflecting the significance of the spatiotemporal relationship, recent research has also demonstrated that the past-behind/future-ahead mapping of time is spontaneously employed when processing temporal constructs [24–26]. Thus, previous studies together suggested that three types of construals (i.e., front-back, up-down, and left-right spatial axes) are recruited in the conceptualization of time, and two types of construals (i.e., up-down and left-right spatial axes) are recruited in the conceptualization of number. Other studies even showed twofold mapping in concept processing within one study [14–15]. For example, Chasteen, Burdzy, and Pratt [27] found faster RTs when targets appeared at compatible locations with the concepts of God (up/right locations) or Devil (down/left locations). This suggests divinity has both vertical and horizontal orientations.

Lakoff and Johnson [1] suggested that many abstract concepts are understood through several external references. Each of these references then provides part of the meaning of the abstract notion. The findings that time, number, and divinity have more than one type of spatial representation are consistent with Lakoff and Johnson’s notion. Kinship, referring to the structure of social relationships, provides another example in that it embodies the attributes that the concepts of *elderliness* and *power* have. The elderliness attribute is mainly reflected in the date of birth (time) and years of age (number). Elder kin were born earlier and junior kin born later, indicating persons older or younger than oneself. The power attribute is mainly reflected in the power of the elder in family life. That is, the elders are more powerful while the juniors are less powerful in a family. As three types of spatial construals are recruited in the conceptualization of time and two types in the conceptualization of number, is it possible that the processing of kinship concept also involves multiple mapping? Moreover, is this spatialization also mediated by the relative Power implied by the kinship terms?

Kinship terms are rich in Chinese culture. For example, father’s elder brother, father’s little brother, the husband of father’s sister, mother’s brother, and the husband of mother’s sister are all called ‘uncle’ in English, while they are called by distinct terms in Chinese: father’s elder brother is called ‘伯父’ (*bofu*), father’s little brother is called ‘叔父’ (*shufu*), the husband of father’s sister is called ‘姑父’ (*gufu*), mother’s brother is called ‘舅父’ (*jiufu*), and the husband of mother’s sister is called ‘姨父’ (*yifu*). Due to the richness and complexity of Chinese kinship terms, the current research used Chinese kinship to investigate the multiple spatial representations in the kinship concept. Spatial metaphors associating filial piety (*xiao*, 孝) with up and down, right and left, as well as front and back are prevalent in Chinese culture, with Chinese often associating up, right, and front with elders and the powerful class while associating down, left, and back with the young and the powerless class. Therefore, it is hypothesized that multiple spatial information would project to the power construct of kinship concept.

To implicitly test the spatial representations of kinship concepts, it is interesting to examine if our mental representations of these concepts affect visual cognition. Recent studies have found participants shifted attention to locations compatible with the concepts of God or Devil [27], affect [7], and mighty [4]. Therefore, the current study investigated the spatial representations of power along three dimensions by requiring participants to complete three different target detection tasks, one in which they responded to targets appearing vertically (above or below the fixation, Experiment 1), another in which targets appeared horizontally (left or right of the fixation, Experiment 2), the other in which targets appeared along the sagittal axis (front or back of the fixation, Experiment 3). Prior to the appearance of the targets, participants were exposed to a cue, which was a word referring either to elder kinship or junior kinship. We predicted that participants would be faster to detect targets that appeared in locations that were spatially compatible to the power of kinship. Moreover, in order to detect the pure effect of power, we statistically controlled the effect of elderliness at step one in regression analysis, as it would be closely related to power in the kinship concept.

In order to avoid a fixed-effect fallacy with regard to language [28], we calculated min *F*’ prime values for each repeated measures ANOVA analysis by controlling for the random effects of Subject and Item simultaneously [28]. The logic is that if min *F'* is significant, then the actual *F'* for their data must be significant, because *F'* must be larger than min *F'*, though min *F'* is criticized to be a somewhat conservative measure. Additionally, in the regression analyses for the RT difference score, we used mixed effects models to control for the random effects of Subject and Item simultaneously [29].

To summarize, if spatial representations along up-down, right-left, and front-back are involved in kinship processing, it is hypothesized that the processing of kinship words has an effect on the shift of attention, resulting in shorter response times for judgment of letters appearing in the congruent part of the screen (elder kin-UP, RIGHT, and FRONT; junior kin-DOWN, LEFT, and BACK). More importantly, if such a result pattern could be predicted by the degree of power of kin after controlling for the effect of elderliness, then it suggests that the activation of spatial representations of kinship along up-down, right-left, and front-back orientation are at least partially attributed to the effect of the power construct.

## Experiment 1: The up-down spatial representation of the power construct

### Participants

Twenty-four undergraduates (11 males, mean age = 20.38± 1.53 years) participated in this experiment for monetary compensation. All were right-handed native speakers of Mandarin Chinese with normal or corrected-to-normal vision. Written informed consent was obtained from each participant. The Institutional Review Board of the South China Normal University (Guangzhou, China) approved this study.

#### Materials

The critical stimuli consisted of 21 elder words (e.g., grandfather) and 21 junior words (e.g., grandson). Before the experiment, all materials were assessed by 28 participants (14 females) who were from the same subject pool but did not contribute to the test data. Assessments were made on the following dimensions: (1) Kinship status: elder, junior, or peer words; (2) Age difference: the absolute year difference between the rater and the kin denoted by kinship words; (3) Degree of power: power difference between S and the kin denoted by the kinship words, ranging from much less to much more powerful than S (1–7 scale); (4) Familiarity: the least to the most familiar (1–7 scale); and (5) Kinship relatedness: the least to the most related (1–7 scale). In the age difference rating and degree of power rating, the raters were first asked to imagine that they themselves had a family member whose status was denoted by the kinship term, and then required to estimate how many years difference between the raters and the kin and the relative power of the kin compared to the raters. For example, a year difference between the rater and grandson of 43 years means that the rater estimated that he/she was 43 years older than his/her grandson. All participants were able to accurately identify the elder or junior characteristics of all kinship words. Estimates of the absolute value of age difference (elder: 40.02 years, junior: 45.08 years; *F*(1,40) = .97, *MSE* = 276.59, *p* = .33, *η*_*p*_^*2*^ = .024) and the absolute difference of power between rater and the kin (elder:1.94, junior: 2.22; *F*(1,40) = 2.65, *MSE* = .32, *p* = .11, *η*_*p*_^*2*^ = .062), and ratings of familiarity (elder: 5.23, junior: 5.02; *F*(1,40) = .38, *MSE* = 1.18, *p* = .54, *η*_*p*_^*2*^ = .009) and kinship relatedness (elder: 4.91, junior: 4.82; *F*(1,40) = .08, *MSE* = 1.04, *p* = .78, *η*_*p*_^*2*^ = .002), showed no statistical difference between elder and junior words. Apart from the experimental items, 8 elder words and 8 junior words were used as practice items.

#### Procedure

The experimental procedure was similar to that of Zanolie et al. [4]. The experiment was conducted in 3 sessions. The first session involved learning to identify target kinship words quickly and accurately. The procedure was as follows. First, a fixation was shown in the center of the monitor for 500 ms, followed by an elder or junior word. Participants were asked to judge whether it was an elder or junior word as swiftly and accurately as possible. Participants made their responses by pressing either a key designated as the ‘elder’ key or the ‘junior’ key on the keyboard. Feedback was given to each of the participant’s reactions. After feedback for 1500ms, the next trial began. There were 16 trials (8 for elder words and 8 for junior words) in this session.

The second session involved participants in familiarizing themselves with the letter judgment task. A fixation was shown in the center of the monitor for 500 ms, followed by a word. Similar to the first session, participants judged whether the word was an elder or junior term by pressing either the ‘elder’ key or ‘junior’ key, with no feedback given (different from the first session). Instead, after a 200 ms blank screen, a horizontally centered letter "*p*" or "*q*" appeared at the top or bottom of the screen (at 75% and 25% of the screen height respectively), about 7 cm either above or below the center (within 5°–7°of visual angle). Participants were asked to judge the letter as quickly and accurately as possible by pressing either a key designated as the ‘*p*’ key or the ‘*q*’ key on the keyboard, with feedback for 1500ms. In this session, only when the accuracy of letter judgment reached as high as 85% were the participants allowed to proceed to the third session. Otherwise, participants were asked to keep practicing until they achieved the 85% criterion. There were 16 trials (8 for ‘*p*’ key and 8 for ‘*q*’ key) in this session.

The third session of the experiment followed a procedure similar to the second session, except for the feedback received after the letter position judgment. If the judgment was correct, there appeared a blank screen for 500 ms; if the judgment was incorrect, there appeared a red “incorrect” warning for 1500 ms. Each word was repeated twice, once followed by a top-positioned letter and once by a bottom-positioned letter. This experimental session included two blocks, with each of the elder and junior words appearing once in each block. Thus, each block had 45 trials, including 3 warm-up trials, 21 elder word trials, and 21 junior word trials. There was a one-minute break between blocks with block orders counterbalanced across subjects. The four key-pressing responses (i.e., elder/junior word judgment and letter *p*/*q* judgment) involved A, S vs. K, L keys on the keyboard and were counterbalanced across participants. Additionally, the combination of A, S vs. K, L keys always remained on the same side (hand). That is, if A and S keys were used to judge the kinship words (e.g., A = junior, S = elder; counterbalanced across participants), then K and L keys were used to judge the letter (e.g., K = p, L = q; counterbalanced across participants), and vice versa. In other words, the kinship word judgment task could be performed with the left hand and letter judgment task could be performed with the right hand, and vice versa.

### Results and Discussion

**Fig 1:**
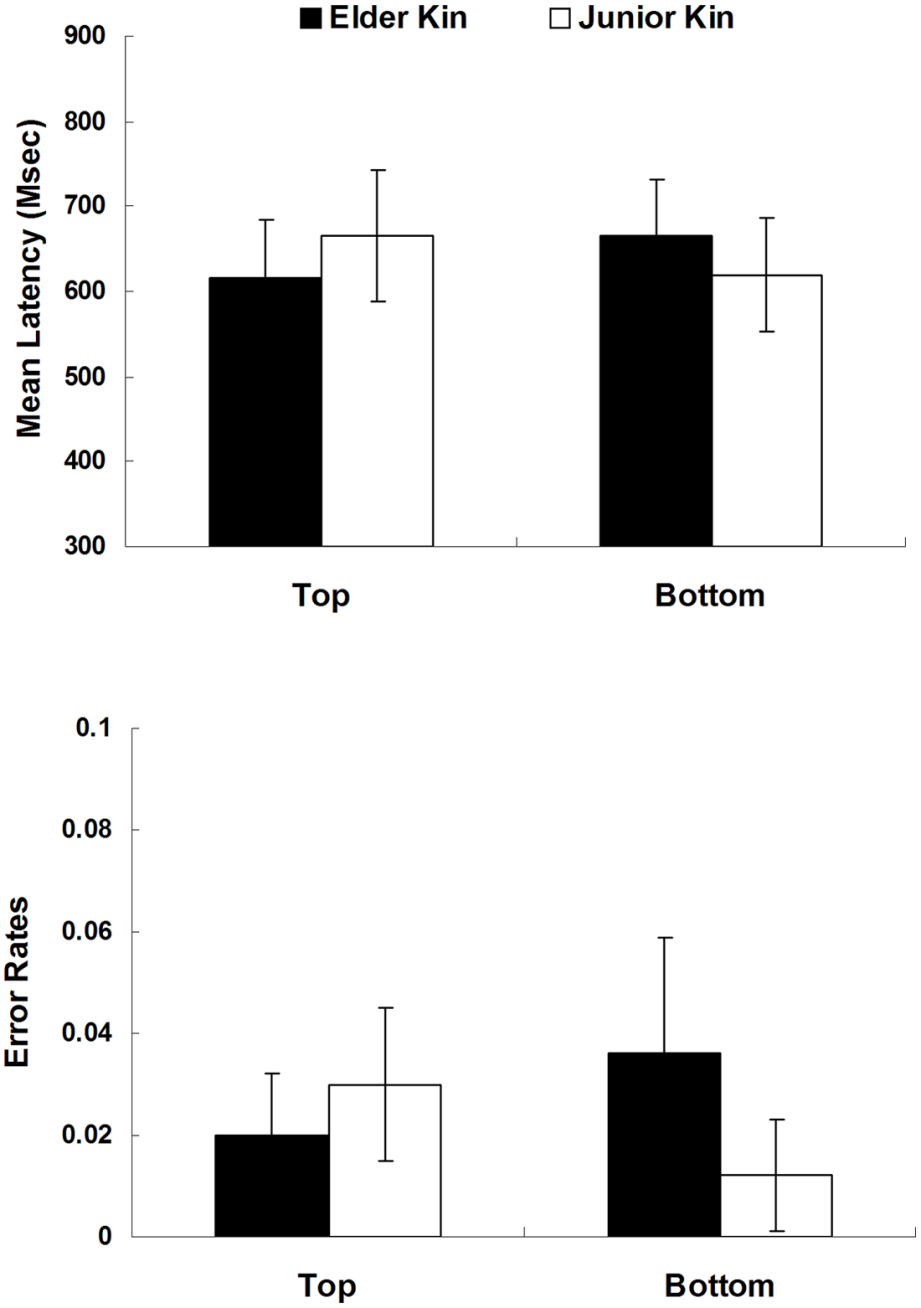
Mean latencies of correct responses (in ms, upper panel) and error rates (%, lower panel) for top letter and bottom letter in each priming condition (elder words and junior words). Error bars represent represent the within-subject 95% confidence intervals.

Prior to the data analysis, outlier data points were removed from the RT and error analysis. Trials with incorrect responses to either the kinship words or to the target letter were rejected from the analysis (3.5%). Trials with reaction times longer than 3000ms to the kinship word or longer than 2000 ms to the letter were excluded from analysis (1.6%). Of the remaining trials, those with reaction times more than two standard deviations faster or slower than the subjects’ condition mean were discarded (4.6%, the raw data of Experiment 1 in S1 File). The RT results (upper panel) and the error rate results (lower panel) are shown in Fig 1.

The reaction times and error scores on the target letter identification task were submitted to 2 (Kin Type: elder vs. junior) × 2 (Letter Position: top vs. bottom) repeated measure analyses of variance (ANOVA) by participants (*F*_*1*_) and by items (*F*_*2*_). In the reaction times there were no main effects of Kin Type, (*F*_*1*_(1,23) = .13, *MSE* = 1021.68, *p* = .73, *η*_*p*_^*2*^ = .005; *F*_*2*_(1,40) = .55, *MSE* = 1290.01, *p* = .46, *η*_*p*_^*2*^ = .01; *min F’*(1,34) = .11, *p* = .74) or Letter Position (*F*_*1*_(1,23) = .03, *MSE* = 1558.85, *p* = .86, *η*_*p*_^*2*^ = .001; *F*_*2*_(1,40) = .003, *MSE* = 2385.08, *p* = .95, *η*_*p*_^*2*^ < .001; *min F’*(1,48) = .003, *p* = .96), but their interaction was significant (*F*_*1*_(1,23) = 81.60, *MSE* = 651.86, *p* < .001, *η*_*p*_^*2*^ = .78; *F*_*2*_(1,40) = 16.80, *MSE* = 2385.08, *p* < .001, *η*_*p*_^*2*^ = .30; *min F’*(1,54) = 13.93, *p* < .001). A decomposition of this interaction showed that letters at the top were discriminated faster when they were preceded by an elder word (616 ms) than when preceded by a junior word (665 ms; *F*_*1*_(1,23) = 26.78, *MSE* = 1092.70, *p* < .001, *η*_*p*_^*2*^ = .54; *F*_*2*_(1,40) = 12.96, *MSE* = 1986.61, *p* = .001, *η*_*p*_^*2*^ = .25; *min F’*(1,63) = 8.73, *p* = .004), whereas letters at the bottom were discriminated faster when they were preceded by a junior word (620 ms) than when preceded by an elder word (665 ms; *F*_*1*_(1,23) = 41.41, *MSE* = 580.84, *p* < .001, *η*_*p*_^*2*^ = .64; *F*_*2*_(1,40) = 8.91, *MSE* = 1688.49, *p* = .005, *η*_*p*_^*2*^ = .18; *min F’*(1,55) = 7.33, *p* = .009).

The analysis of the error scores revealed no significant effects of Kin Type or Letter Position (*ps* > .10), while their interaction was significant (*F*_*1*_(1,23) = 4.54, *MSE* = .002, *p* = .044, *η*_*p*_^*2*^ = .17; *F*_*2*_(1,40) = 5.72, *MSE* = .001, *p* = .022, *η*_*p*_^*2*^ = .13; *min F’*(1,54) = 2.53, *p* = .11). A decomposition of this interaction revealed that letters at the bottom were responded to with fewer errors when they were preceded by a junior word (1.2%) than when preceded by an elder word (3.6%, *F*_*1*_(1,23) = 3.83, *MSE* = .002, *p* = .062, *η*_*p*_^*2*^ = .14; *F*_*2*_(1,40) = 6.58, *MSE* = .001, *p* = .014, *η*_*p*_^*2*^ = .14; *min F’*(1,48) = 2.42, *p* = .13), whereas when the letter was presented at the top there was no difference in error rate between the two types of kinship words (*F*_*1*_(1,23) = 1.50, *MSE* = .001, *p* = .23, *η*_*p*_^*2*^ = .06; *F*_*2*_(1,40) = .87, *MSE* = .001, *p* = .36, *η*_*p*_^*2*^ = .02; *min F’*(1,63) = .55, *p* = .46).

**Fig 2:**
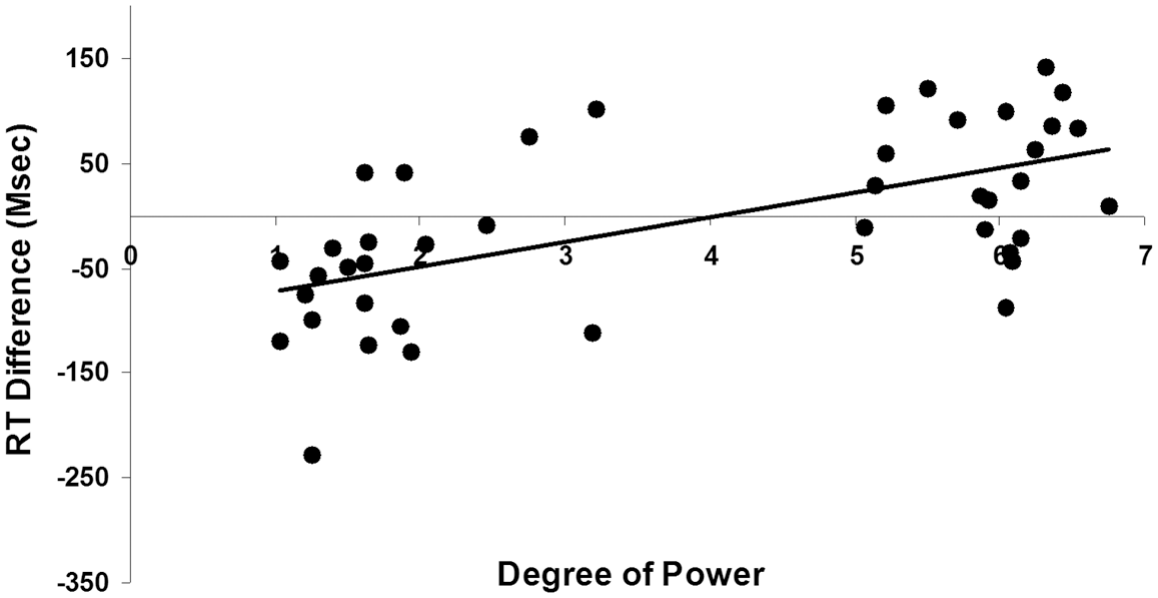
Degree of power predicts RT difference scores in the Experiment 1 (the vertical spatialization).

In order to further demonstrate that the degree of power may uniquely result in the spatialization found in kinship word processing we calculated the difference in response time between bottom (letter located at the bottom of the screen) and top conditions (letter located at the top of the screen) (i.e., bottom condition minus top condition). We then utilized regression to model degree of power as a predictor of RT difference scores with kin type and degree of elderliness (i.e., the relative age difference between subjects and a potential kin; for example, a grandfather would have a value of +40 and a grandson -40) as control variables. We also controlled for the random effects of subjects and items. The results showed that the degree of power had a significant unique effect on the RT difference scores (*b* = 31.15, *t*(821) = 2.09, *p* = .037, *R* ^2^ = .059; Fig 2), while the significant effect of degree of elderliness (*b* = .91, *t*(822) = 1.97, *p* = .049, *R* ^2^ = .054) became non-significant (*b* = .51, *t*(821) = 1.03, *p* = .30) after entering degree of power into the regression equation.

The results of Experiment 1 showed that after controlling for the effect of elderliness, the power construct embedded in the concept of kinship could uniquely activate an underlying vertical spatial image schema, thus affecting vertical spatial attention. As participants processed power, their attention shifted in an image schema congruent direction (powerful-top or powerless-bottom), thereby facilitating identification of targets in the corresponding location, which is consistent with the findings of previous studies [4].

## Experiment 2: The left-right spatial representation of the power construct

### Method

A new group of 31 participants from the same subject population as in Experiment 1 was recruited in this experiment (12 males, mean age = 20.13 ± 1.65 years). Written informed consent was obtained from each participant. The Institutional Review Board of the South China Normal University (Guangzhou, China) approved this study. The design, stimuli, and the procedure were all identical to those in Experiment 1 except that (1) the position of the letter *p* or *q* was located to the left (vertically centered and horizontally 25% of the screen width) or to the right (vertically centered and horizontally 75% of the screen width) of the fixation, about 9 cm either to the left or the right of the center (within 7°–9°of visual angle); (2) to avoid the confounding effect of response code, the four key-pressing responses were changed to D, C vs. K, M keys on the keyboard and counterbalanced across participants. Additionally, the combination of D, C vs. K, M keys always remained on the same side (hand). That is, if D and C keys were used to judge the kinship words (e.g., D = junior, C = elder; counterbalanced across participants), then K and M keys were used to judge the letter (e.g., K = p, M = q; counterbalanced across participants), and vice versa. In other words, the kinship word judgment task could be performed with the left hand and the letter judgment task could be performed with the right hand, and vice versa.

### Results and discussion

**Fig 3:**
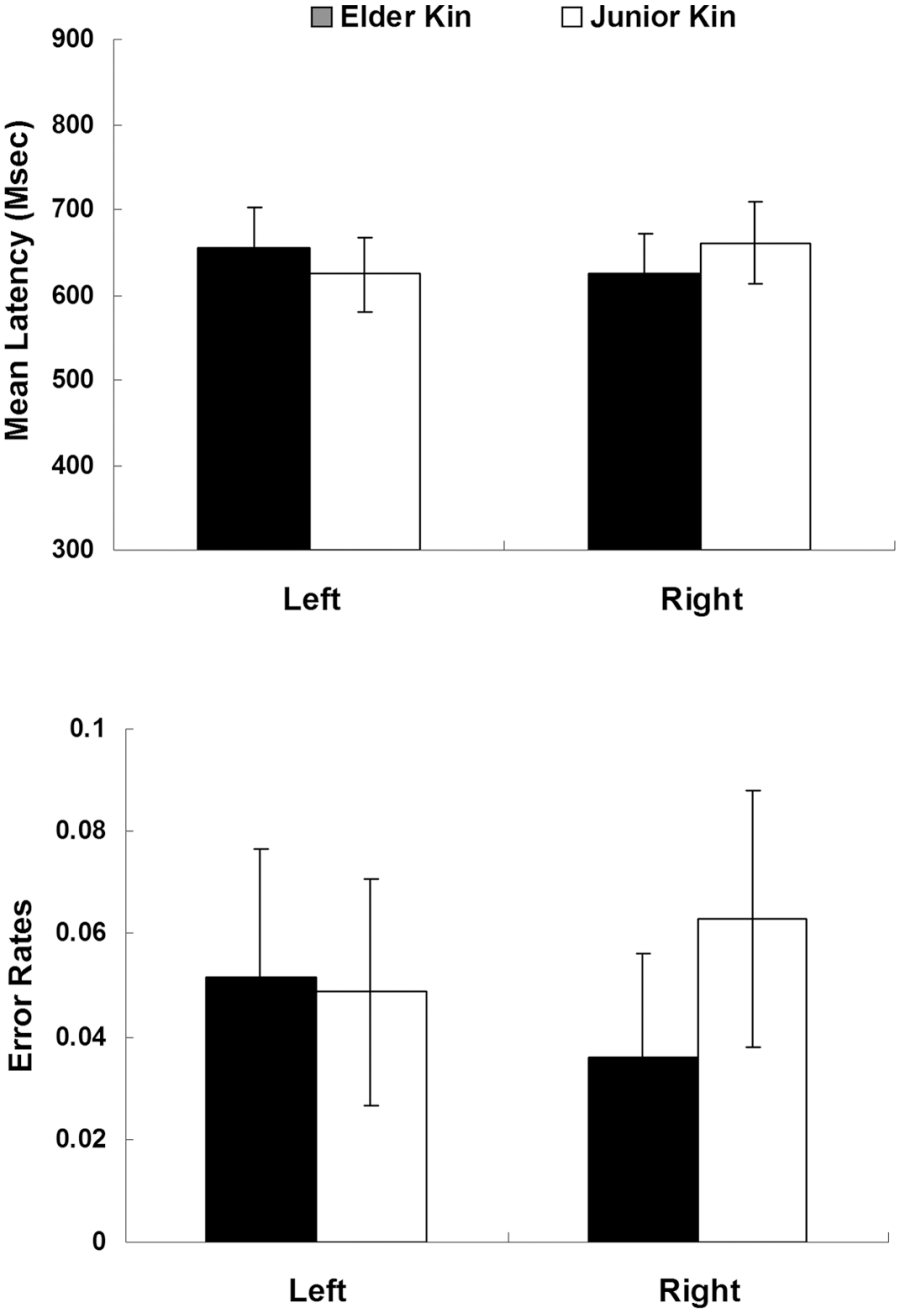
Mean latencies of correct responses (in ms, upper panel) and error rates (%, lower panel) for left letter and right letter in each priming condition (elder words and junior words). Error bars represent the within-subject 95% confidence intervals.

As in Experiment 1, prior to the data analysis, trials with incorrect responses to either the kinship word or to the letter were rejected, accounting for 6.5%. Trials with reaction times longer than 3000 ms to the kinship word or longer than 2000 ms to the letter were excluded from analysis, making up 1.8% (the raw data of Experiment 2 in S2 File). Of the remaining trials, trials with reaction times that exceeded two standard deviations above or below each participant’s condition mean were eliminated from the RT analyses, making up 6.1%. The RT results (upper panel) and the error rate results (lower panel) are shown in Fig 3.

Data analysis was conducted in the same way as Experiment 1 using 2 (Kin Type: elder vs. junior word) × 2 (Letter Position: left vs. right) repeated measures ANOVAs by participants and by items. There were no significant main effects for either Kin Type (*F*_*1*_(1,30) = .03, *MSE* = 2349.36, *p* = .87, *η*_*p*_^*2*^ = .001; *F*_*2*_(1,40) = .33, *MSE* = 749.07, *p* = .57, *η*_*p*_^*2*^ = .008; *min F’*(1,35) = .03, *p* = .86) or Letter Position (*F*_*1*_(1,30) = .21, *MSE* = 1452.49, *p* = .65, *η*_*p*_^*2*^ = .007; *F*_*2*_(1,40) < .001, *MSE* = 1205.18, *p* = .99, *η*_*p*_^*2*^ < .001; *min F’*(1,40) = .001, *p* = .98). An interaction between the two factors was significant (*F*_*1*_(1,30) = 22.60, *MSE* = 1559.19, *p <* .001, *η*_*p*_^*2*^ = .43; *F*_*2*_(1,40) = 17.50, *MSE* = 1205.18, *p* < .001, *η*_*p*_^*2*^ = .30; *min F’*(1,70) = 9.86, *p* = .002). A decomposition of this interaction showed that response time to left letters was shorter when they were preceded by a junior word (624 ms) than by an elder word (656 ms) (*F*_*1*_(1,30) = 8.41, *MSE* = 1920.80, *p* = .007, *η*_*p*_^*2*^ = .22; *F*_*2*_(1,40) = 10.15, *MSE* = 1274.24, *p* = .003, *η*_*p*_^*2*^ = .20; *min F’*(1,66) = 4.60, *p* = .036), whereas response time to right letters was shorter when they were preceded by an elder word (625 ms) than by a junior word (660 ms, *F*_*1*_(1,30) = 9.63, *MSE* = 1987.75, *p* = .004, *η*_*p*_^*2*^ = .24; *F*_*2*_(1,40) = 12.35, *MSE* = 680.01, *p* = .001, *η*_*p*_^*2*^ = .24; *min F’*(1,65) = 5.41, *p* = .023). The analysis of the error scores revealed no significant effect (*ps >* .05).

**Fig 4:**
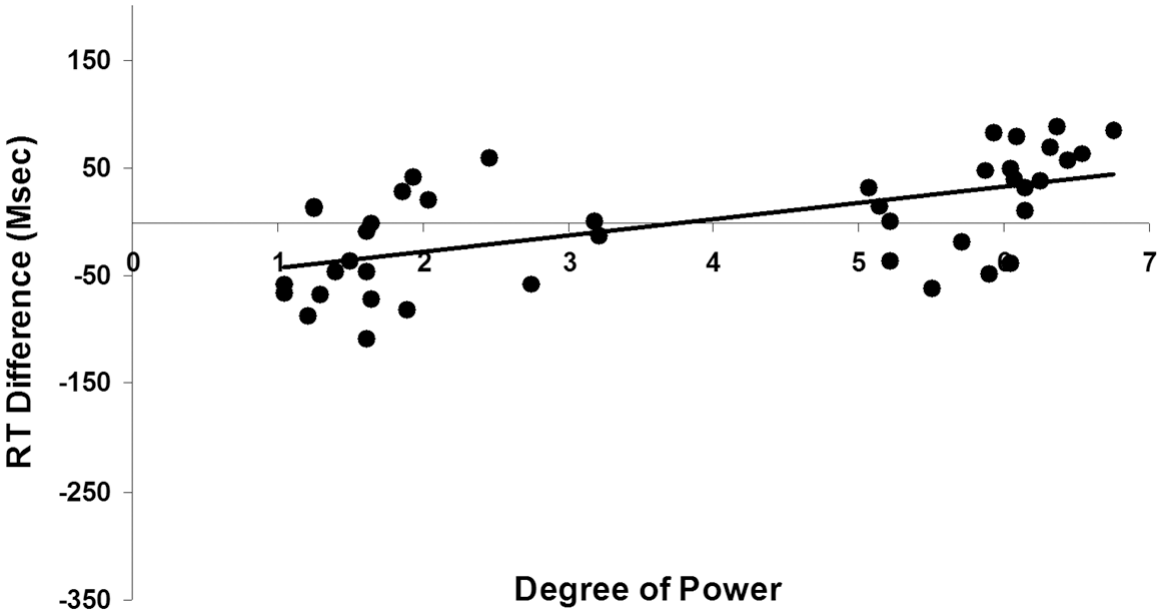
Degree of power predicts RT difference scores in the Experiment 2 (the left-right horizontal spatialization).

Similar to Experiment 1, we utilized regression to model degree of power as a predictor of RT difference scores (i.e., left condition minus right condition) with kin type and degree of elderliness as control variables. We also controlled for the random effects of subjects and items. The results showed that the degree of power had a significant unique effect on the RT difference scores (*b* = 28.86, *t*(954) = 2.01, *p* = .045, *R* ^2^ = .024; Fig 4), while the significant effect of degree of elderliness (*b* = .90, *t*(955) = 2.07, *p* = .039, *R* ^2^ = .020) became non-significant (*b* = .51, *t*(954) = 1.07, *p* = .29) after entering degree of power into the regression equation. The results of Experiment 2 showed that after controlling for the effect of elderliness, the power construct embedded in the concept of kinship could uniquely activate an underlying left-right spatial image schema, and thus affect left-right horizontal spatial attention.

## Experiment 3: The front-back spatial representation of the power construct

### Method

**Fig 5:**
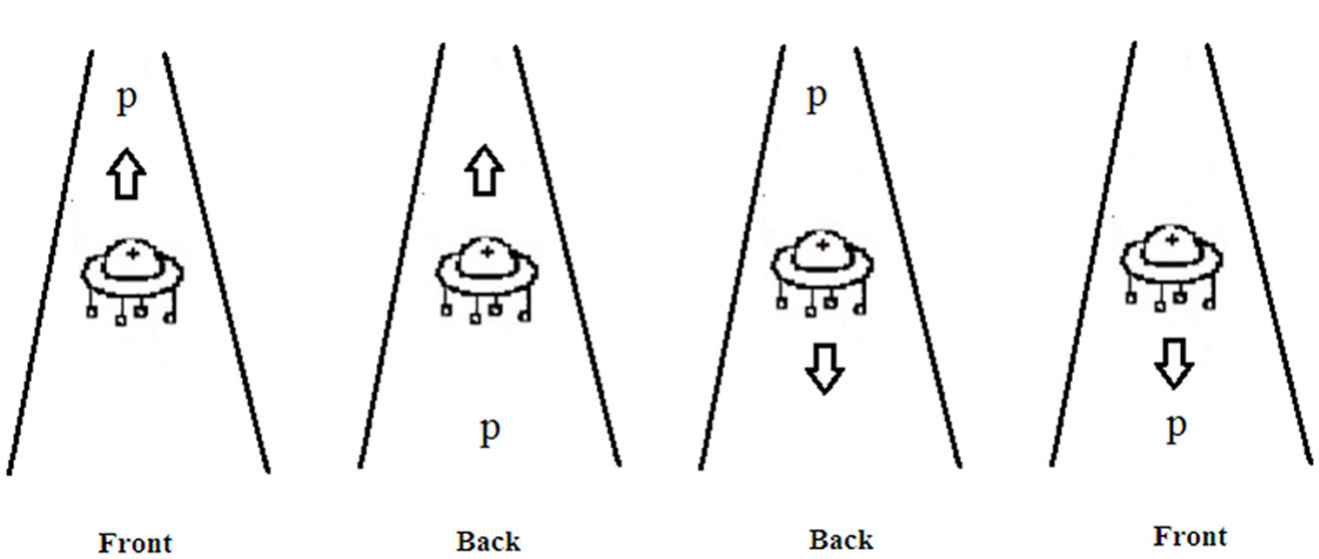
Schematic of the spatial letters used in Experiment 3.

Another new group of 23 participants from the same subject population as in Experiments 1 and 2 was recruited in this experiment (8 males, mean age = 20.35 ± 1.11 years). Written informed consent was obtained from each participant. The Institutional Review Board of the South China Normal University (Guangzhou, China) approved this study. The design, stimuli, and the procedure were identical to those in Experiment 1 except (1) after the identification of kinship words, a moving object with a fixation and an arrow appeared in the center of the computer screen (see Fig 5). These stimuli are adapted from an original stimulus set constructed by Boroditsky [30]; (2) 200 ms later a horizontally centered letter (*p* or *q*) was presented at the top or bottom of the computer screen (at 75% or 25% of the screen height respectively), about 7 cm either above or below the cross (within 5°–7°of visual angle) with the object remaining onscreen; (3) in order to balance the moving direction of the object, the experimental session of the current experiment included four blocks, with each of the elder and junior words appearing once in each block.

### Results and discussion

**Fig 6:**
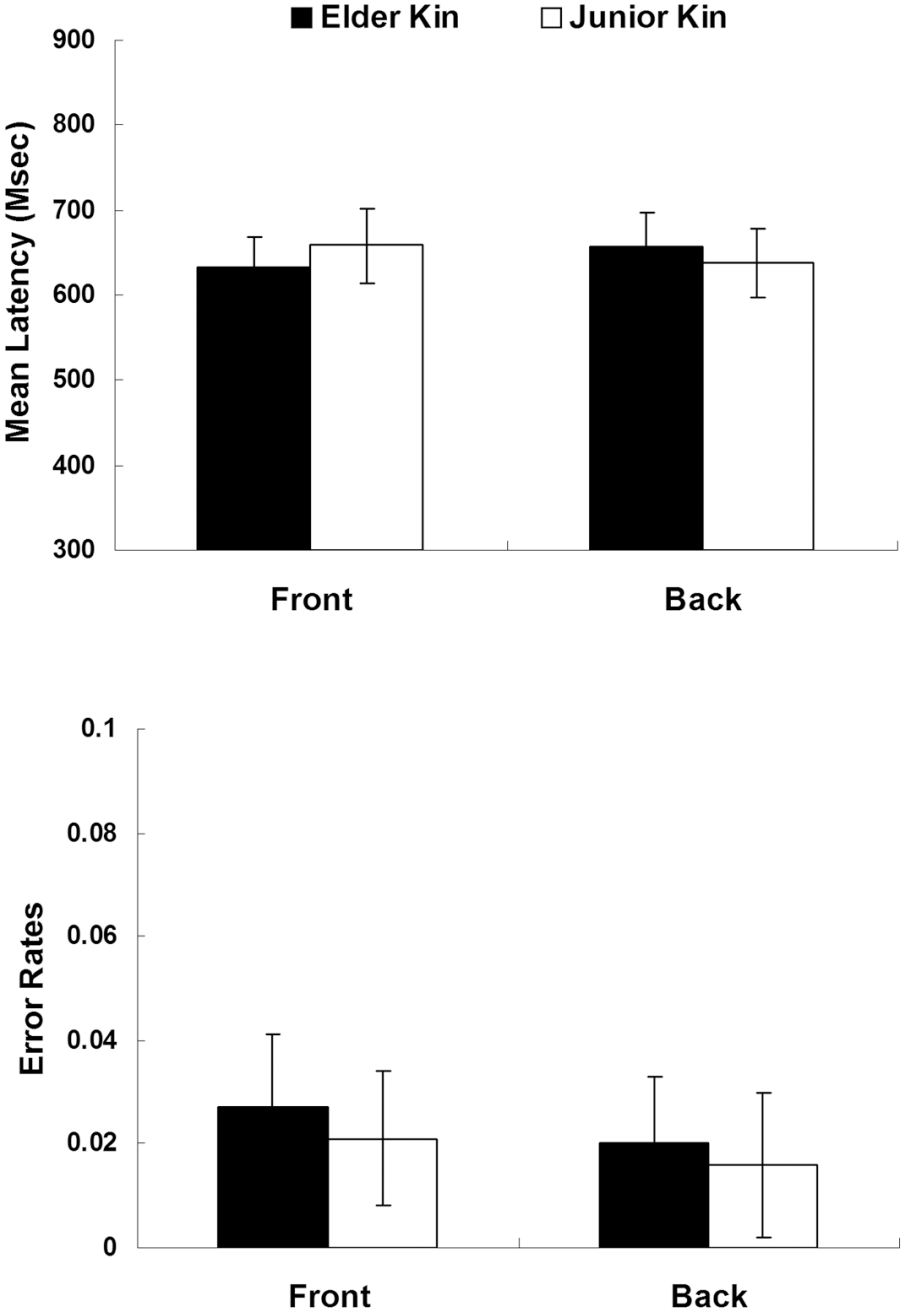
Mean latencies of correct responses (in ms, upper panel) and error rates (%, lower panel) for letter in the front of the moving object and letter at the back of the moving object in each priming condition (elder words and junior words). Error bars represent the within-subject 95% confidence intervals.

As in Experiments 1 and 2, prior to the data analysis, trials with incorrect responses to either the kinship word or to the letter were rejected, accounting for 7.3%. Trials with reaction times longer than 3000 ms to the kinship words or longer than 2000 ms to the letter were excluded from analysis, making up 1.2%. Of the remaining trials, trials with reaction times that exceeded two standard deviations above or below each participant’s condition mean were eliminated from the RT analyses, making up 4.4% (the raw data of Experiment 3 in S3 File). The RT results (upper panel) and the error rate results (lower panel) are shown in Fig 6.

As in Experiment 1, two-way repeated measures ANOVAs were performed for the RTs and error rates. In regard to the RTs, no significant main effects for Kin Type (*F*_*1*_(1,22) = .34, *MSE* = 774.38, *p* = .57, *η*_*p*_^*2*^ = .02; *F*_*2*_(1,40) = .28, *MSE* = 463.87, *p* = .60, *η*_*p*_^*2*^ = .007; *min F’*(1,60) = .15, *p* = .70) or Letter Position were found (*F*_*1*_(1,22) = .18, *MSE* = 504.82, *p* = .68, *η*_*p*_^*2*^ = .008; *F*_*2*_(1,40) = .29, *MSE* = 683.31, *p* = .59, *η*_*p*_^*2*^ = .007; *min F’*(1,48) = .11, *p* = .74). An interaction between these two factors was significant (*F*_*1*_(1,22) = 27.15, *MSE* = 401.60, *p* < .001, *η*_*p*_^*2*^ = .55; *F*_*2*_(1,40) = 16.25, *MSE* = 683.31, *p* < .001, *η*_*p*_^*2*^ = .29; *min F’*(1,62) = 10.17, *p* = .002). Simple effect analysis showed that when the letter was in the front of the moving object, it took less time to discriminate the letter when it was primed by an elder word (633 ms) than by a junior word (658 ms)(*F*_*1*_(1,22) = 12.73, *MSE* = 570.47, *p* = .002, *η*_*p*_^*2*^ = .37; *F*_*2*_(1,40) = 12.18, *MSE* = 559.49, *p* = .001, *η*_*p*_^*2*^ = .23; *min F’*(1,57) = 6.22, *p* = .016), while when the letter was at the back of the moving object, it took less time to discriminate the letter when primed by a junior word (638 ms) than by an elder word (657 ms) (*F*_*1*_(1,22) = 6.44, *MSE* = 605.51, *p* = .019, *η*_*p*_^*2*^ = .23; *F*_*2*_(1,40) = 7.52, *MSE* = 587.68, *p* = .009, *η*_*p*_^*2*^ = .16; *min F’*(1,54) = 3.47, *p* = .067). There was no significant effect in error rate analysis (*ps >* .10).

**Fig 7:**
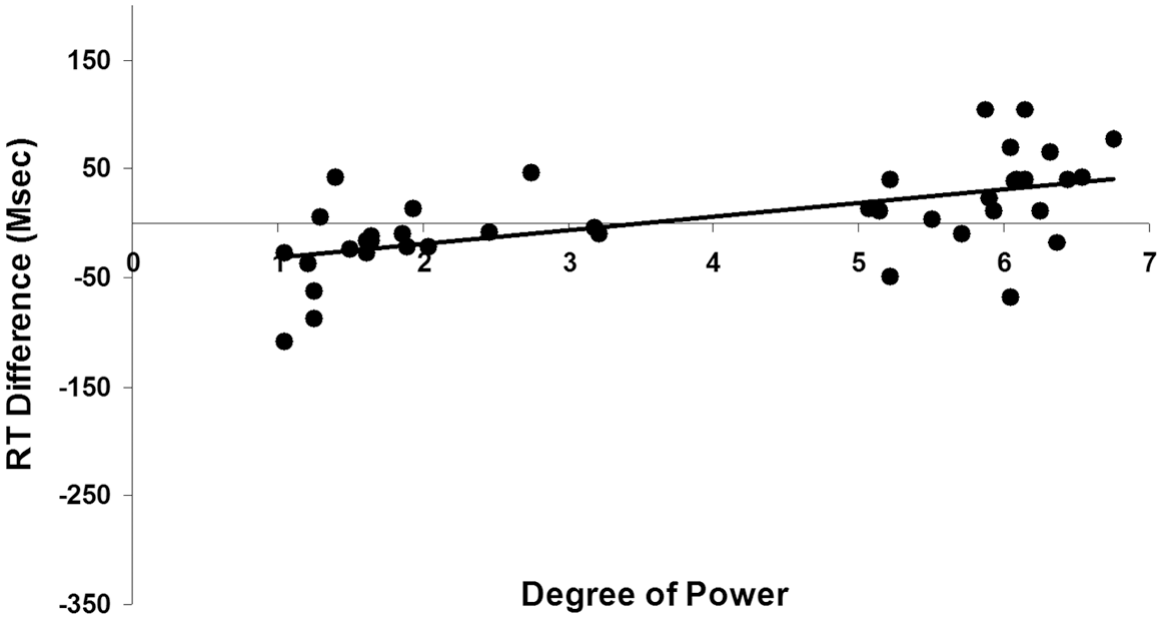
Degree of power predicts RT difference scores in the Experiment 3 (the front-back horizontal spatialization).

Similar to Experiment 1, we utilized regression to model degree of power as a predictor of RT difference scores (i.e., back condition minus front condition) with kin type and degree of elderliness as control variables. We also controlled for the random effects of subjects and items. Results showed that degree of power had a significant unique effect on the RT difference scores (*b* = 23.79, *t*(939) = 2.38, *p* = .017, *R* ^2^ = .034; Fig 7), while the significant effect of degree of elderliness (*b* = .74, *t*(940) = 2.39, *p* = .017, *R* ^2^ = .028) became non-significant (*b* = .40, *t*(939) = 1.19, *p* = .23) after entering degree of power into the regression equation. The results of Experiment 3 showed that after controlling for the effect of elderliness, the power construct embedded in the concept of kinship could uniquely activate the underlying front-back spatial image schema, and thus affect front-back horizontal spatial attention.

## General Discussion

Our motivating research question concerned the multiple spatial representations in the kinship concept. The results indicated that targets that were located in higher or lower regions of the visual field, left or right regions of the visual field, and front or back of a particular object in the visual field were identified more quickly, when they were congruent with a semantic term (e.g., an elder word or a junior word) that preceded the visual search. The regression results further confirmed that these shifts of attention along up-down, right-left, and front-back dimensions in external space were uniquely attributable to the power construct embedded in the kinship concept, but not the numerical or temporal meaning of each of the kinship terms, suggesting the presence of vertical, left-right horizontal, and front-back horizontal spatial representations in the power construct.

These results are consistent with the metaphoric structuring view that metaphors act to set up correspondences between conceptual structures of the target and base concepts, suggesting something important about the structure of our conceptual system [30]. There is evidence from studies that have investigated the processing of abstract concepts through reference to others (i.e., metaphorical mapping) suggesting the existence of multiple external concrete categories in the domain of abstract categories [31]. Many abstract concepts are found to be understood through several such references. Each of these references then provides part of the meaning of the abstract notion [1]. Thus, the importance of activating the multiple spatial representations in power processing is consistent with the above finding that abstract concepts could be understood through several concrete references.

A strong valuation for kin and lineage exists throughout Chinese history and has been noted in much anthropological literature [32]—a valuation in which the imperatives of filial piety (*xiao*) are considered regulators of cross-generational and cross-status relationships. Filial piety derives from that most fundamental human bond: parent and child. The Chinese word for this is *xiao* (孝). The top portion of the character for *xiao* shows an old man and underneath, a young man supporting the old man. There are different interpretations of the meaning of the character: the burden and oppression by the old of the young, the support by the young of the older generation, and the respect of the young for the older generation [33]. Obviously, Chinese family hierarchy was emphatically symbolized in the concept of *xiao*, which is more accurately rendered “filial subordination.” When wills clashed, it was expected that the will of a family superior (i.e., elder kin) should prevail over the will of a family inferior (i.e., junior kin). Traditional law held a child’s insubordination to a parent to be a capital offense, and a daughter-in-law's insubordination to her parents-in-law grounds for divorce. That is, elder kin are powerful in a family, while junior kin are powerless. As filial piety means parental power, it is reasonable that power is central to the spatialization of kinship.

Is it possible that our results are also due to other concepts implied by kinship that are spatialized (e.g., number and time)? It is obvious that the elderliness attribute of a kin is reflected in the date of birth (time) and years of age (number). The temporal aspect of elderliness would predict that powerful is linked with “left” and “behind” while powerless “right” and “front,” as in many languages the past (e.g., seniority) is “left” and “behind,” and the future (e.g., Juniority) is “right” and “ahead.” However, de la Fuente, Santiago, Román, Dumitrache, and Casasanto [34] found that Arabic speakers tend to conceptualize the future as “behind” and the past as “ahead of” them, demonstrating that the space-time mappings in people’s minds are conditioned by their cultural attitudes toward time. This result suggests that culture could shape spatial representations of a concept. Under the influence of Chinese culture (Chinese often associate up, right, and front with elders and the powerful class while down, left, and back are associated with the young and the powerless class), it is sensible that powerful could be conceptualized as “right” and “ahead” and powerless as “left” and “behind”, which is different from the prediction based on time.

Additionally, the numerical aspect of elderliness would predict a pattern in which powerful (seniority, larger number) is correlated with right and possible front, whereas powerless (juniority, small number) is correlated with left and possible back based on the SNARC effect. Chinese culture emphasizes that everybody should respect persons elder than oneself. Our results that powerful is conceptualized as up, right, and front and powerless as down, left, and back would indicate such a close association between the numerical aspects of elderliness and power. The current study used relative age difference to indicate degree of elderliness. That is, we focused on the numerical aspect of the kinship concept and tried to separate the spatial representations of number and power in kinship concept.

According to Dehaene et al. [20], the SNARC effect reflects a space-related representation of numerical magnitudes (*mental number line*) with a genuine left-to-right orientation. More recently, studies using the SNARC paradigm have also found evidence for a vertical effect where small and large numbers are located towards the bottom and top of space, respectively [15]. Our finding that junior kin oriented attention to the left and downward while elder kin oriented attention to the right and upward, appears to be a SNARC-like effect as kin also indicate numerical magnitude (i.e., elderliness). The current study found that after controlling for the effect of elderliness, power could significantly and uniquely predict the shifts of attention along up-down, right-left, and front-back dimensions, while the significant effect of elderliness became non-significant after the entering of power. Such results suggested that the spatial representations activated during kinship terms processing would not be an effect of elderliness (SNARC-like effect), nor a mixed effect of elderliness and power. In other words, what we found in the current study is a pure effect of power, indicating something important about the power and status denoted by kinship.

We note that in other linguistic domains—quantification and individuation [35–36], verbs [37], and spatial terms [38]—there is clear evidence that different languages capture different regularities in the world and cue speakers of different languages in different ways [39]. Therefore, it is possible that the results obtained in the present set of experiments are culture-specific and would not be observed in a language in which the concept of kin did not imply power and social status. In Chinese culture, the way to express *xiao* (filial piety) is typically to see elderly people in front of, above, or to the right of junior people. Thus, it is possible that the understanding of the power construct embedded in Chinese kinship may also emerge directly from our sensory experience of up/down, left/right, and front/back. Such these speculations, while interesting, will require more evidence.

Our results showed that the speed of response to a letter presented at the top of the screen (+polar endpoint) is facilitated when it is preceded by a high power kinship word (+polar endpoint) compared to a low power kinship word (-polar endpoint), whereas the speed of response to a letter presented at the bottom of screen (-polar endpoint) is facilitated when it is preceded by a low power kinship word (-polar endpoint) compared to a high power kinship word (+polar endpoint). A similar result pattern was also found in left-right horizontal and front-back horizontal dimensions. According to the polarity interpretation offered by Proctor and Cho [11], these compatibility effects found in the current study would be elicited by response codes due to an overlap between the power and both the target location and response side. However, this is unlikely since the p and q response keys in Experiments 1 and 3 were located horizontally, and the response keys in Experiment 2 were located vertically. Thus, our results could not be explained by the effect of polarity correspondence (i.e, stimulus-response code mapping) on classification tasks. Additionally, the current study investigated three types of spatial representations separately with one in each experiment. Thus, our results could not provide evidence to support the notion that the power construct activates three separate spatial associations simultaneously.

To conclude, in line with previous findings, results of the present study indicate that spatial representation is involved in the processing of concepts such as power embedded in kinship. Moreover, such activation is not oriented along one dimension but three dimensions, namely, up-down, left-right, and front-back. This is the first study to demonstrate the multiple spatial representations of the power construct.

## Supporting Information

S1 File The raw data of Experiment 1. (ZIP)

S2 File The raw data of Experiment 2. (ZIP)

S3 File The raw data of Experiment 3. (ZIP)
